# Supplementary material for: Pulmonary Vein Isolation Outcome Degree Is a New Score for Efficacy of Atrial Fibrillation Catheter Ablation
Source: J Clin Med. 2021 Dec 13;10(24):5827. doi: 10.3390/jcm10245827 (PMC8708501; doi:10.3390/jcm10245827)
Supplement: Supplementary file 1 [file jcm-10-05827-s001.zip › jcm-1416822-SI.pdf]

Figure S1(A)

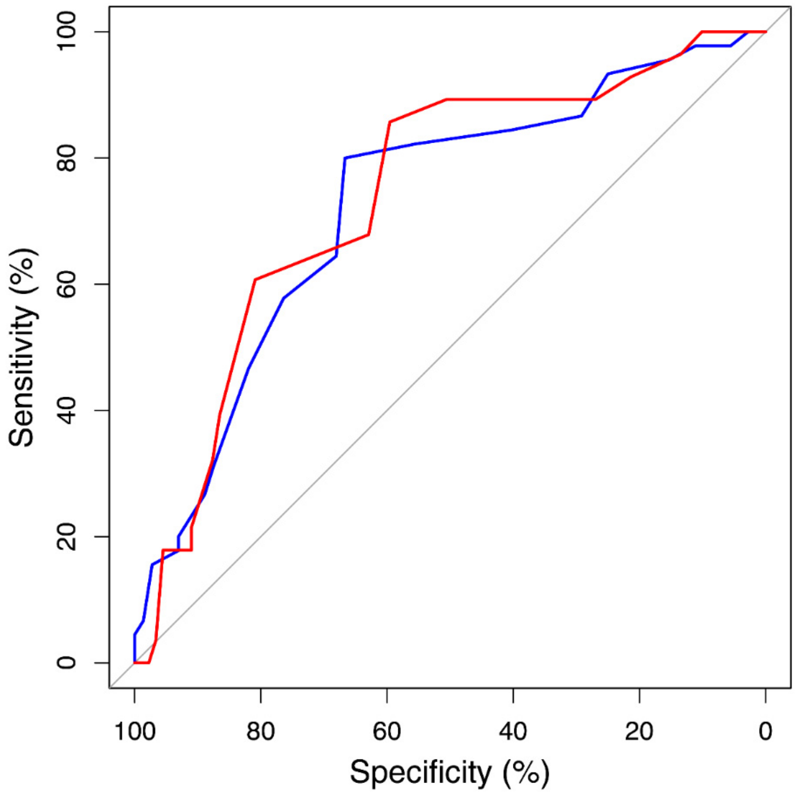

Figure S1(B)

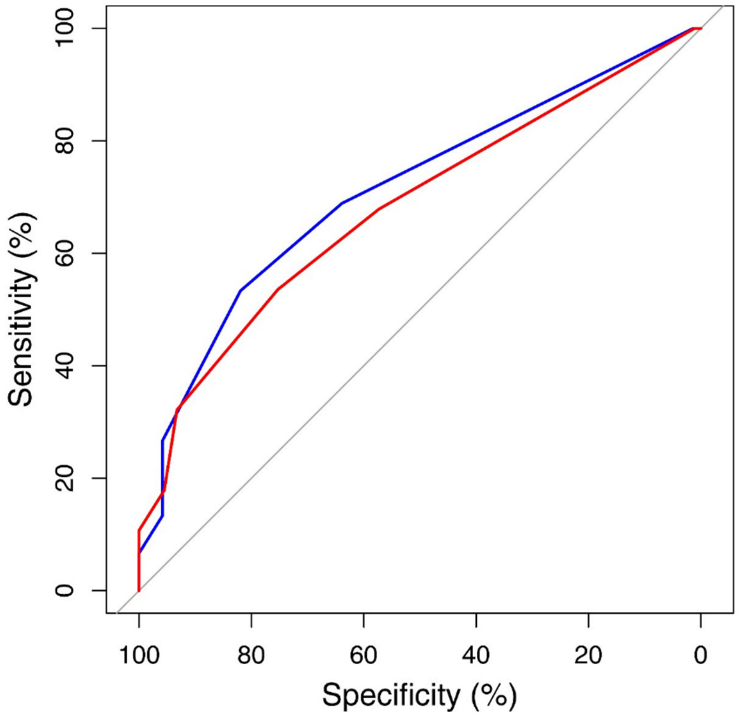

Figure S1(C)

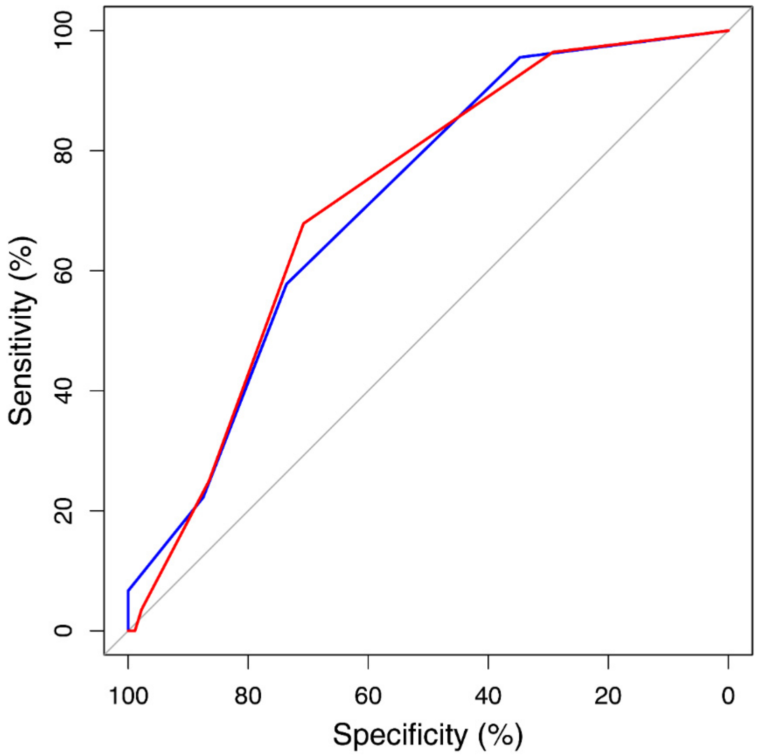

Figure S1. Receiver operating characteristic (ROC) curves for left atrial diameter (**A**), LVEF (**B**) and CHA_2DS_2-VASc score (**C**). Blue curve: procedural success compared with procedural failure; red curve: procedural and clinical success compared with procedural and clinical failure.
